# Letter to the Editor: The rise of ChatGPT: exploring its potential in vaccine hesitancy

**DOI:** 10.1097/JS9.0000000000001115

**Published:** 2024-01-17

**Authors:** Shidong Wang, Yujiao Wu

**Affiliations:** a Department of Respiratory Medicine, Shaoxing Second Hospital, Zhejiang; b Department of Pharmacy, Changzhou Third People’s Hospital, Changzhou Medical Center, Nanjing Medical University, Jiangsu, People’s Republic of China

*Dear Editor*,

A series of successful SARS-CoV-2 vaccines have played a huge role in the efforts to control the COVID-19 pandemic, and saved at least tens of millions of lives. However, during the immunisation procedure for COVID-19, vaccine hesitancy has surfaced, especially in vulnerable populations like patients and pregnant women^1^.

We have read recent articles published by Nazir *et al*.^2^ and Suvvari *et al*.^3^ in *International Journal of Surgery*. They note that vaccine hesitancy among pregnant women and infants may be prevalent in respiratory syncytial virus (RSV) vaccinations, and that one of the major reasons for the Measles virus (MeV) outbreak is vaccine hesitancy. It is striking that vaccine hesitancy is such a widespread phenomenon.

Recent breakthroughs in artificial intelligence have resulted in the development of highly complex chatbot technologies, particularly ChatGPT, developed by OpenAI, which has garnered a lot of attention. The field of large language models (LLMs) is experiencing rapid development, especially in the field of global health^4^. Some views suggest that chatGPT has the potential to alleviate vaccine hesitancy by effectively addressing widespread concerns surrounding vaccines, including but not limited to adverse reactions, long-term safety implications, and the duration of vaccine development. By providing individuals with comprehensive information and instructions, chatGPT can effectively dispel doubts and facilitate the formation of informed decisions. However, ChatGPT currently also has certain issues. The most serious problem is that compared to search engines, we are unable to find the source of information for ChatGPT. This means that the accuracy of the answers provided by ChatGPT may be questionable^5^. Therefore, ChatGPT can serve as a helpful friend providing suggestions, rather than assuming the role of a medical authority in decision-making for the user.

Clinicians do not have enough energy or time to educate patients about vaccine-related concerns in clinical practice due to limited medical resources and other factors. ChatGPT can help in this area. This is particularly significant in developing countries, where medical resources may be scarce. ChatGPT provides not just answers to questions, but also provides a portion of emotional value. While there are certain limitations, the LLMs represented by ChatGPT may add a new dimension to the resolution of vaccine apprehension.

This study was supported by the Science and Technology Project of Changzhou (Grant No. CJ20239024).

## Ethical approval

Not applicable.

## Consent

Not applicable.

## Sources of funding

Not applicable.

## Author contribution

S.W.: writing; Y.W.: study design.

## Conflicts of interest disclosure

There are no conflicts of interest.

## Research registration unique identifying number (UIN)

Not applicable.

## Guarantor

Yujiao Wu.

## Data availability statement

Not applicable.

## Provenance and peer review

Not commissioned, externally peer-reviewed.
